# Chimera of Globin/Nitric Oxide Synthase: Toward Improving Nitric Oxide Homeostasis and Nitrogen Recycling and Availability

**DOI:** 10.3389/fpls.2020.575651

**Published:** 2020-09-30

**Authors:** Fiorella Del Castello, Andrés Nejamkin, Noelia Foresi, Lorenzo Lamattina, Natalia Correa-Aragunde

**Affiliations:** Instituto de Investigaciones Biológicas, Consejo Nacional de Investigaciones Científicas y Técnicas (CONICET), Universidad Nacional de Mar del Plata - CONICET, Mar del Plata, Argentina

**Keywords:** cyanobacteria, globin, nitric oxide synthase, nitrogen use efficiency, nitrogen recycling, plants

## Introduction

As a result of anthropogenic activities, there are regions suffering extreme climate changes (hot temperatures, droughts, floods), that generate serious and harmful environmental and socio-economic consequences (Hoegh-Guldberg et al., 2018). Climate change has a strong impact on agriculture, mainly by increasing soil degradation and reducing land productivity (Olsson et al., 2019). Desertification decreases soil macronutrients as organic carbon (OC), phosphorus (P) and nitrogen (N) (Shang et al., 2013; Tang et al., 2015). Considerable reductions of OC, P and N are caused by a decline in soil water, vegetation and wind erosion. It is estimated that when soil water is less than 30%, OC and N decrease approximately 50% (Shang et al., 2013). The intensity and frequency of extreme climate events predicted will increase the competition for nutrients, notably N among plants and soil microorganisms (Bennett and Klironomos, 2019; Pugnaire et al., 2019).

N availability is essential for net primary production and determines changes in total vegetation biomass and soil OC (Tharammal et al., 2019). Approximately 150 Tg/yr of N is spilled to the land surface as a result of industrial activities and fossil fuel combustion (Schlesinger, 2009). Huge amounts of N fertilizers are used to increase crop productivity, but only 25%–30% are retained in plant biomass (Nadelhoffer et al., 1999; Schlesinger, 2009). The excess of N is then transported to aquatic environments resulting in eutrophication and reduction of dissolved O_2_, percolated to the groundwater or loss to the atmosphere, increasing greenhouse gas (GHG) emissions such as nitrogen oxides (NOx) (Schlesinger, 2009; Breitburg et al., 2018). In this context, bio/technological solutions like genetic modification of crops are required to avoid, reduce and reverse GHG emissions and water eutrophication, contributing to climate change mitigation. New biotechnological strategies developed for the agriculture to increase N use efficiency (NUE) in crops would help to fulfill this purpose. In this opinion, we will discuss some studies of nitric oxide (NO) synthases (NOS) and nitrate reductase (NR) enzymes playing a collaborative role with globin (Hb) proteins, leading to NO and/or nitrate (NO_3_^-^) homeostasis in different organisms. We propose that chimeric globin-NOS such as the NOS from *Synechococcus* PCC 7335 (SyNOS) may have evolved in photosynthetic microorganisms contributing to a more efficient N recycling and sustaining growth in N fluctuating conditions. Engineered crops that maximize NUE would result in a better adaptation to changing climatic conditions with less N fertilization, preserving aquatic ecosystems and atmosphere.

## Nitric Oxide/Nitrate Homeostasis by the Association of Nitric Oxide Synthase and Globin

NOS enzymes catalyze the oxidation of Arginine (Arg) to produce citrulline and NO, a molecule that participates in diverse processes ranging from growth and development and stress responses (Alderton et al., 2001; Crane et al., 2010; Del Castello et al., 2019). Mammalian NOSs have a N-terminal oxygenase domain (NOSoxy) and a C-terminal reductase domain (NOSred) joined by a calmodulin binding motif (Griffith and Stuehr, 1995). In Gram positive bacteria, NOS have only one domain and consist of NOSoxy similar to that of animals (Crane et al., 2010). NOS proteins were also identified in photosynthetic microorganisms such as green algae, diatoms and cyanobacteria (Foresi et al., 2010; Di Dato et al., 2015; Kumar et al., 2015). While a NOS-like activity has been detected in plant extracts (Corpas and Barroso, 2017), the search in more than 1000 sequenced transcriptomes suggests that this gene is not present in terrestrial plants (Jeandroz et al., 2016; Foresi et al., 2017). This is intriguing, and raises the question of whether, in terrestrial plants, NOS has not been searched correctly, or simply is not present. Particularly, the characterization of a NOS in the cyanobacterium *Synechococcus* PCC 7335 (SyNOS) opens new and interesting questions regarding its biological function since it is a chimera of a globin domain joined to a canonical NOS (Correa-Aragunde et al., 2018).

Globins (Hbs) are heme-proteins comprising three families, flavohemoglobin (FHb), globin-coupled sensors and truncated-Hb (THb). The main function of Hbs is related to NO detoxification by a NO dioxygenase (NOD) activity oxidizing NO to NO_3_^-^ (Vinogradov and Moens, 2008). The interaction of NO molecule and Hbs was studied in bacteria, animals and plants (Poole and Hughes, 2000; Seregélyes and Dudits, 2003; Tejero and Gladwin, 2014). However, the current knowledge shows that there are few examples of a coupled reaction between NOS enzymes and Hb and leading to NO/NO_3_^-^ release regulation. In mammals, endothelial NOS (eNOS) associates with α-Hb to modulate NO diffusion during vascular smooth muscle relaxation. α-Hb binds to eNOS and oxidates NO to NO_3_^-^, preventing that NO reaches smooth muscle cells (Straub et al., 2012). The interaction between NOS and Hbs to regulate NO homeostasis has also been reported in bacteria. *Staphylococcus aureus* generates energy using NO_3_^-^ as an alternative electron acceptor in O_2_ limiting conditions and it is principally regulated by NOS and HMP (bacterial FHb) (Kinkel et al., 2016). Under micro-aerobic conditions, NOS-derived NO inhibits aerobic respiration by interacting with cytochrome oxidases. Under aerobic conditions, NO consumption predominates by HMP activity providing a homeostatic mechanism that modulates respiration in response to O_2_ availability (Kinkel et al., 2016).

## Hbs and NO Participation in N Metabolism in Photosynthetic Organisms

More interestingly, the interaction of Hbs and NO has a remarkable role in the N metabolism of photosynthetic organisms. N metabolism was largely studied in higher plants, green algae and diatoms (Flynn, 1991; Coruzzi and Zhou, 2001; Bertoni, 2017). Once NO_3_^-^ is internalized, it is reduced to NO_2_^-^ by NR, the first enzyme in the assimilation pathway (Wilkinson and Crawford, 1991). In *Chlamydomonas*, NO_2_^-^ is the substrate of NOFNiR (NO Forming NO_2_^-^ Reductase) that catalyzes NO production even in the presence of high (millimolar) concentrations of NO_3_^-^, which inhibits NO production by the NO_2_^-^ reductase activity of NR. NOFNiR together with NR are intimately connected both at transcriptional and posttranscriptional regulatory levels suggesting enzyme cooperativity (Chamizo-Ampudia et al., 2016; Llamas et al., 2017). Furthermore, NO_3_^-^ controls the expression of THbs by regulating transcription factors activity. THB1 generates a direct connection between NO and NO_3_^-^ metabolism by modulating NO levels and NR activity. NR is also able to reduce THB1, through its diaphorase activity. Interestingly this activity is more efficient than others reducing cofactors like NADH, FAD, or cytochrome b5 reductase which possesses high homology to NR (Sanz-Luque et al., 2015).

The interaction between NR and THB1 in N metabolism has also been demonstrated in diatoms. Smith et al. (2019) show that two of the strongest NO_3_^–^ sensitive genes were part of the Hb superfamily with high homology to THB1 from *Chlamydomonas*. Considering these evidences, the interaction NR-THB1 seems to work as a complex for NO and NO_3_^-^ homeostasis. Scavenging of NO to produce NO_3_^-^, maximizes NR efficiency during N assimilation diminishing N loss (as gaseous NO), improving N utilization in photosynthetic organisms.

## Chimeric Globin-NOS From *Synechococcus* PCC 7335 and Its Potential Role in N Metabolism

The NOS from *Synechococcus* PCC 7335 (SyNOS) was the first NOS characterized that contains a globin domain (Correa-Aragunde et al., 2018). Biochemical analysis showed that SyNOS produces NO and NO_3_^-^ *in vitro* in an approximately 1:3 ratio and data suggest that the globin domain is mainly responsible for NO_3_^-^ production (Picciano and Crane, 2019). Chimeric globin-NOS similar to SyNOS appears in some diatom and cyanobacteria genomes (Di Dato et al., 2015; Correa-Aragunde et al., 2018).

Heterologous expression of SyNOS in *Escherichia coli* allows bacteria to improve growth under limiting N conditions. Furthermore, SyNOS expression may contribute the use of Arg as a N source in bacteria (Correa-Aragunde et al., 2018). In cyanobacteria and plants, Arg serves as a major organic N storage and transport. Thus, Arg metabolism plays a key role in N distribution and remobilization in these organisms (Winter et al., 2015). Arg dihydrolase, another novel enzyme recently characterized, converts Arg to ornithine, CO_2_ and ammonium (Zhang et al., 2018; Burnat et al., 2019). Results indicate that Arg dihydrolase is part of a mechanism involved in N storage and remobilization in cyanobacteria conferring rapid adaptability to N fluctuations (Zhang et al., 2018). N fluctuation in aquatic environments, mainly rivers, lakes, estuaries and marine coastal regions, are result of precipitation events, droughts, wind velocity and warming affecting water-sediment interactions and anthropogenic N inputs (Conley et al., 2009; Havens et al., 2016). We predict that fluctuations in N availability will become more severe in a faster and unpredictable climate change.

The characterization of novel functional genes is significant for the understanding of the mechanisms governing the adaptation to changing environmental conditions. Chimeric genes provide an exceptional source of new cell functions, resulting in organisms that may adapt to different environments by developing new metabolic skills. The characterization of the chimera globin-NOS like SyNOS could break a paradigm on NOS biological functions. In our opinion, SyNOS represent a sophisticated evolution of NOS genes with the ability to regulate the NO/NO_3_^-^ homeostasis and signaling, besides the potential function in N metabolism.

## SyNOS Expression as a Biotechnological Strategy to Improve NUE in Plants

Early attempts to increase NUE and yield in crops were based in manipulating NO_3_^-^ transporters or N assimilation genes, but negative or contradictory results suggest that is a complex agronomic trait (Li et al., 2017). NR overexpression affected intracellular NO_3_^-^ levels but did not increase yield nor improve plant growth (Ferrario-Méry et al., 1998; Djennane et al., 2002). More promising results have been achieved by manipulating genes involved in plant N remobilization (Oliveira et al., 2002; Gaufichon et al., 2017; Chen et al., 2019). Specially, the manipulation of genes involved in amino acid catabolism and transport seems to be good strategies to remobilize organic N storage increasing seed filling and plant yield (Good et al., 2007; Shrawat et al., 2008; Ma et al., 2013; Meng et al., 2015; Perchlik and Tegeder, 2017).

In this opinion, we propose that the expression of the chimeric globin-NOS from *Synechococcus* PCC 7335 could improve cellular N metabolism enhancing NUE and crop production under N shortage conditions. There are already evidences of NOS from different origins (animals and algae), recombinantly expressed in plants (Rodríguez-Ruiz et al., 2019). In all cases, the NOSs expressed are active in the transgenic plants displaying high NO levels and stress tolerance (Chun et al., 2012; Shi et al., 2012; Shi et al., 2014; Cai et al., 2015; Foresi et al., 2015). SyNOS as well as animal NOS function with tetrahydrobiopterin (BH_4_) cofactor and not with tetrahydrofolate (THF) (Picciano and Crane, 2019), the main known pterin in plants (Sahr et al., 2005). However, plants can enzymatically produce dihydrobiopterin (BH_2_), and it was reported that the photosynthetic apparatus can reduce pterins from dihydro to tetrahydro form (Fuller and Nugent, 1969). Otherwise, it is known that other pterins can fulfill NOS activity (Presta et al., 1998). Recently, Nejamkin et al. (2020) have shown that the expression of the NOS from the algae *Ostreococcus tauri* (OtNOS) in tobacco increases growth rate, number of flowers and seed yield, and that this phenotype is lost when plants are grown in low N condition.

**Figure 1:**
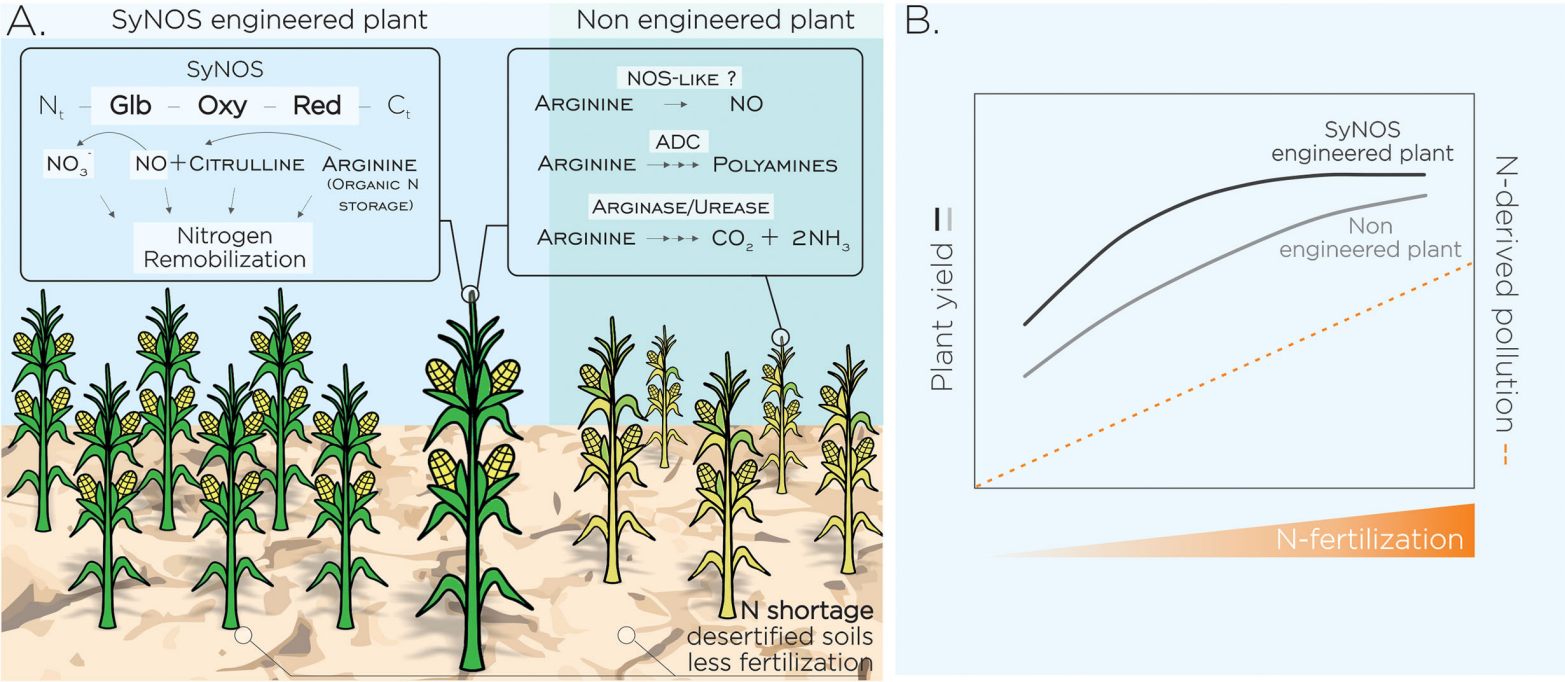
Plant N remobilization as a target of biotechnological strategy to promote lower fertilization and N pollution in a climate change context. **(A)** Increased N remobilization from plant reservoirs through heterologous and/or overexpression of specific enzymes could enhance growth and yield of crops to cope with desertified soils. Plants tolerant to N scarcity will also help to reduce the use of fertilizers and their impact on the environment. We postulate the heterologous expression of NOS from *Synechococcus* PCC 7335 (SyNOS) in plants as a biotechnological tool to increase N remobilization. SyNOS contains a singular globin (Glb) domain at the 5’-end, followed by the canonical oxygenase (Oxy) and reductase (Red) domains of NOS enzymes. SyNOS activity may remobilize N from Arg internal pools in plants leading to NO and NO_3_^-^ formation, the last contributes to improve plant NUE. SyNOS would be a gain of function increasing the number of Arg-metabolizing enzymes in plant cells: arginase, Arg decarboxylase (ADC) and NOS-like activity. **(B)** Postulated scenario showing non-engineered plant yield (adapted from Brueck and Lammel, 2016) and putative yield of SyNOS-engineered plants, in variable levels of N fertilization and environmental pollution. Dashed lines show positive correlation between environmental pollution and N fertilization (Zhang et al., 2019).

Engineered plants that express SyNOS unlike the expression of other NOS would remobilize N stored in Arg internal pools producing NO/NO_3_^-^ and may be an interesting tool to improve N metabolism (**Figure 1**). Although plants have Arg degrading enzymes (i.e., arginase), the incorporation of Arg catabolic pathways in crops may improve N remobilization and plant productivity. It was reported that the overexpression of arginases improves NUE and seed yield in rice and cotton under different N conditions (Ma et al., 2013; Meng et al., 2015). These results support our hypothesis and suggest that SyNOS expression would be an alternative strategy that generates other N sources (NO_3_^-^/NO) and may favor crop productivity. NO produced by SyNOS might also cope to stress tolerance (Del Castello et al., 2019; Kolbert et al., 2019). Despite this, unexpected phenotypes concerning changes in NO homeostasis should be analyzed, as well as the consequences of potential changes in Arg concentration as substrate of different metabolic pathways (i.e., polyamine synthesis). In addition, genetic variations among cultivars, time of harvest (vegetative/reproductive phase), crop management should be considered when defining a strategy for NUE improvement.

Overall, the discovery of novel enzymes involved in NUE in photosynthetic organisms will facilitate transferring our knowledge from theory to practice and generate crops to boost agriculture when confronting serious environmental challenges.

## Author Contributions

FC and AN contributed equally to this Hwork. All authors contributed to the article and approved the submitted version.

## Funding

This research was supported by the Agencia Nacional de Promoción Científica y Tecnológica (ANPCyT: PICTs 2927/2015 to LL and PICT 2524/2018 to NC-A.), the Consejo Nacional de Investigaciones Científicas y Técnicas (CONICET, PIP 0646/2015 to NC-A) and institutional grants from the Universidad Nacional de Mar del Plata (UNMdP), Argentina.

## Conflict of Interest

The authors declare that the research was conducted in the absence of any commercial or financial relationships that could be construed as a potential conflict of interest.
